# MCHB-DETR: An Efficient and Lightweight Inspection Framework for Ink Jet Printing Defects in Semiconductor Packaging

**DOI:** 10.3390/mi17010109

**Published:** 2026-01-14

**Authors:** Yibin Chen, Jiayi He, Zhuohao Shi, Yisong Pan, Weicheng Ou

**Affiliations:** 1 School of Mechanical and Electrical Engineering, Guangdong University of Technology, Guangzhou 510006, China; 2112301497@mail2.gdut.edu.cn (Y.C.); addone0801@163.com (J.H.); 2112201415@mail2.gdut.edu.cn (Z.S.); 19924686553@163.com (Y.P.); 2 Experimental Teaching Department, Guangdong University of Technology, Guangzhou 510006, China

**Keywords:** semiconductor packaging, inkjet defect, transformer based detector, lightweight detection

## Abstract

In semiconductor packaging and microelectronic manufacturing, inkjet printing technology is widely employed in critical processes such as conductive line fabrication and encapsulant dot deposition. However, dynamic printing defects, such as missing droplets and splashing can severely compromise circuit continuity and device reliability. Traditional inspection methods struggle to detect such subtle and low-contrast defects. To address this challenge, we propose MCHB-DETR, a novel lightweight defect detection framework based on RT-DETR, aimed at improving product yield in inkjet printing for semiconductor packaging. MCHB-DETR features a lightweight backbone with enhanced multi-level feature extraction capabilities and a hybrid encoder designed to improve cross-scale and multi-frequency feature fusion. Experimental results on our inkjet dataset show a 29.1% reduction in parameters and a 36.7% reduction in FLOPs, along with improvements of 3.1% in mAP@50 and 3.5% in mAP@50:95. These results demonstrate its superior detection performance while maintaining efficient inference, highlighting its strong potential for enhancing yield in semiconductor packaging.

## 1. Introduction

In semiconductor packaging and heterogeneous integration for next-generation electronic devices, inkjet printing is playing an increasingly critical role. Its high precision and digitally controlled additive nature make it particularly suitable for performing fine-scale tasks such as high-density interconnect fabrication and photoresist coating [1,2,3]. However, the process is highly susceptible to disturbances such as nozzle clogging and fluctuations in jetting parameters, which can lead to micron-scale defects that are difficult to detect visually. At the semiconductor scale, these defects are no longer minor process irregularities and may directly cause fatal failures such as open circuits, short circuits between adjacent lines, or drift in device parameters, thereby posing a significant threat to product yield and reliability [4,5]. Consequently, achieving real-time and accurate defect detection for inkjet printing on production lines is essential for enabling its large-scale deployment in high-value semiconductor manufacturing.

Accurately detecting such defects presents multiple challenges: the defects are typically micron-scale, exhibit irregular shapes, and are often concealed within complex backgrounds characterized by significant noise or low contrast. Traditional manual inspection is time-consuming, labor-intensive, and highly subjective, making it unsuitable for large-scale manufacturing environments [6]. Meanwhile, conventional image-processing methods that rely on handcrafted features often struggle to capture complex or subtle defect patterns and are highly sensitive to variations in illumination and image noise [7]. These limitations severely constrain the broader adoption of inkjet printing in modern industrial settings where high precision and efficiency are critical, particularly in semiconductor manufacturing.

Recent advances in computer vision and deep learning have introduced data-driven solutions for defect detection. In particular, object detection methods based on convolutional neural networks (CNNs), such as R-CNN [8], SSD [9], and the YOLO series [10,11,12,13,14], have significantly addressed the limitations of traditional approaches that rely on handcrafted features. By enabling automated feature extraction and hierarchical representation of both local textures and global structures, these methods overcome the rigidity and poor generalization of rule-based techniques and have been widely applied in defect classification and localization tasks [15,16,17,18]. However, CNN-based detectors still face challenges when dealing with small, dense, or low-contrast defects due to their reliance on local receptive fields [19,20]. Additionally, their dependence on bounding box regression and non-maximum suppression (NMS) often leads to missed detections in dense object scenarios [21,22].

To address these issues, recent studies have explored Transformer-based detectors, such as the Detection Transformer (DETR). Developed by Facebook AI, DETR eliminates handcrafted components traditionally used in object detection, such as anchor boxes and NMS, and achieves end-to-end detection through global modeling capabilities [23]. However, DETR suffers from slow convergence and high computational complexity due to its deep encoder–decoder structure. To mitigate these drawbacks, Deformable DETR [24] introduced a multi-sampling attention mechanism known as Deformable Attention to accelerate model convergence. Conditional DETR V2 [25] highlighted the importance of query initialization in DETR and instead used the encoder for query initialization. Group DETR [26] employed grouped object queries and a one-to-many dynamic label assignment strategy to significantly enhance performance and convergence speed. Nevertheless, the high computational demands of these models continue to hinder their applicability in real-time detection scenarios. RT-DETR [27], combining a hybrid encoder with an IoU-aware query selection module, achieves a balance between accuracy and computational efficiency, making it the first real-time, end-to-end Transformer-based object detection algorithm.

However, directly applying RT-DETR to inkjet printing defect detection still presents several challenges. Traditional backbone networks, such as ResNet, alleviate the vanishing gradient problem by introducing residual connections, enabling the construction of deeper architectures and improved feature representation. However, these deep models are often associated with increased computational complexity and parameter overhead, which may limit their applicability in industrial inspection scenarios with strict real-time and resource constraints. Moreover, although global self-attention mechanisms can model long-range dependencies, their homogeneous attention tends to overlook heterogeneous frequency information, which is critical for organizing multi-frequency features effectively.

To address these limitations, we propose MCHB-DETR, a lightweight cross-scale feature fusion detection model based on Transformer architecture, specifically designed for inkjet printing defect detection. Our approach builds upon RT-DETR and introduces key enhancements to both the backbone network and the encoder to improve cross-scale and cross-frequency feature modeling.

The main contributions of this work are summarized as follows:

1. To optimize the detection performance under inkjet defects, an efficient lightweight component MELAN was designed. By emphasizing feature representation and multi-level fusion, the sensitivity of the network to defect related areas can be enhanced.
2. By combining Coordinate Attention (CA) and Haar wavelet decomposition, the CH-ADown module has been implemented. This module enables the model to focus on defect areas in complex backgrounds and preserve key feature information.
3. By introducing HiLo-AIFI module and BSSFF module, an enhanced hybrid encoder integrating multi frequency and cross scale feature fusion is developed. This architecture enhances the robustness of the model in scale variations and complex backgrounds.
4. The performance of the method was validated on self-built inkjet datasets and publicly available X-ray datasets. The results indicate that our model outperforms state-of-the-art detectors in mAP and model complexity while maintaining high inference speed, confirming its practicality in various industrial detection scenarios.

The remainder of this paper is organized as follows. Section 2 reviews related work relevant to this study. Section 3 describes the data collection process for the inkjet dataset and provides a detailed overview of the proposed MCHB-DETR model architecture. Section 4 presents the experimental setup and offers a comprehensive analysis of the results. Finally, Section 5 concludes the paper.

## 2. Related Works

### 2.1. Inkjet Printing Defects Detection Based on Deep Learning

Currently, deep learning-based object detection frameworks have achieved remarkable success across a wide range of industrial applications, including steel surface inspection [28], semi-conductor manufacturing [29], and infrastructure monitoring [30]. These methods benefit from end-to-end learning capabilities and automatic feature extraction, making them well-suited for complex and dynamic environments. Among them, CNN-based approaches, particularly the YOLO series, are widely favored due to their real-time performance, end-to-end training pipeline, and robust multi-scale feature representations. In recent years, the growing number of related publications has demonstrated their increasing impact. For instance, Xiang et al. [31] enhanced YOLOv8 by integrating block-wise feature fusion convolutions and multi-dimensional convolutional cooling attention modules, as well as incorporating auxiliary training heads. Their approach achieved strong performance in common industrial surface defect detection tasks. Jia et al. [32] improved YOLOv5 by integrating deformable convolutions and coordinate attention mechanisms, thereby reducing model complexity while enhancing the detection accuracy of bearing surface scratches.

With the continuous advancement of deep learning, researchers have begun exploring its applications in inkjet printing defect detection—a field characterized by small target sizes, complex backgrounds, and challenging imaging conditions. Applications in this domain generally fall into two categories. The first focuses on adjusting process parameters during the inkjet printing operation to optimize print quality. For example, Choi et al. [33] employed YOLOv5 to quantify various process parameters in synchronous multi-nozzle inkjet spraying, demonstrating the feasibility of autonomous real-time process monitoring in large-scale electronic manufacturing. Zhang et al. [34] integrated a convolutional neural network (CNN) with a vision camera system for in situ process monitoring and printing status evaluation, enabling effective anomaly detection in the aerosol jet printing (AJP) process. The second category primarily targets product quality inspection at the post-printing stage, aiming to ensure reliability in mass production. Yao et al. [35] proposed a flexible electronics defect detection model based on YOLOv5, which leverages neural architecture search to enhance model performance. Liu et al. [36] introduced a lightweight YOLOv5 model for label defect detection in inkjet printing, integrating C3-DCN modules and RepConv structures, and applied pruning techniques to reduce computational costs.

Although these studies demonstrate the feasibility of deep learning in inkjet applications, it is noteworthy that most are adaptations of general-purpose detection models, lacking architectural innovations specifically tailored to the unique characteristics of inkjet defects. Compared with more mature fields such as steel surface inspection and semiconductor defect detection, inkjet defect detection remains relatively underexplored, with limited open datasets, benchmark evaluations, and task-specific model designs. This gap highlights the urgent need for customized, lightweight, and robust solutions capable of effectively addressing the fine-grained and dynamic nature of inkjet printing defects.

### 2.2. Detection Methods Based on Transformer

The Transformer architecture was originally proposed for natural language processing tasks, and due to its ability to model long-range dependencies and capture global context, it has recently gained significant attention in the field of computer vision. Vision Transformers (ViT) and related models have demonstrated highly competitive performance across various image classification, segmentation, and detection tasks, often outperforming traditional CNN-based methods when trained on large-scale datasets [37].

These advancements have promoted the application of Transformer-based detection frameworks in industrial defect detection. For example, Wu et al. [38] proposed a real-time Transformer-based model for detecting surface defects on compressor blades. Their approach combines an improved ResNet18 backbone with a hierarchical multi-scale feature pyramid network and utilizes an Inner-GIoU loss function, achieving fast inference speed and high accuracy. Ge et al. [39] introduced the HPRT-DETR model, which integrates deformable attention mechanisms, feature concatenation and cascade fusion modules, and a normalized Wasserstein distance (NWD) metric loss. This framework addresses challenges such as small defects, complex backgrounds, and low detection accuracy in metal bipolar plate inspection. Cheng et al. [40] developed the Adln-DETR model for insulator defect detection, combining Gaussian saliency-guided GSG adapters for convolutional feature modulation with foresight-capable LFO adapters, achieving competitive results even on relatively small datasets.

Despite these advancements, several key limitations remain. On one hand, existing methods largely rely on standard backbones such as ResNet, which are not specifically optimized for detecting small, irregularly shaped defects and lack frequency-aware or cross-scale feature fusion mechanisms. These capabilities are critical in complex industrial defect scenarios. Furthermore, the inherent global self-attention mechanism in Transformers exhibits quadratic computational complexity with respect to the spatial dimensions of feature maps, which significantly constrains their efficiency when applied to high-resolution images.

Given that inkjet printing defect detection involves small-scale, texture-mixed, and irregularly shaped targets, the global receptive field and flexible feature representation capabilities of Transformers make them particularly well-suited for this domain. However, the application of Transformer-based detection models in inkjet printing remains largely unexplored. Most existing studies focus on more traditional industrial surfaces and lack customized architectures tailored to the specific visual challenges posed by inkjet defects. These gaps highlight the need for task-specific Transformer frameworks that are not only accurate but also computationally efficient. Such models must be capable of multi-scale feature fusion, robust representation under noisy conditions, and real-time inference on high-resolution data.

## 3. Materials and Methods

This section presents the collection and preparation process of the inkjet defect dataset, employing various data augmentation techniques to address the limitations in representing dynamic process defects and complex background coverage within the dataset. To overcome the suboptimal performance of existing Transformer-based detectors in inkjet printing defect detection, this paper proposes a novel object detection model, MCHB-DETR, which is an improved version of the RT-DETR model. The architecture of the proposed method is then detailed, highlighting its effectiveness in enhancing detection performance and robustness under the aforementioned challenges.

### 3.1. Inkjet Datasets

In this study, the inkjet printing process follows a drop-on-demand mechanism commonly employed in semiconductor packaging for functional material deposition. The defects in this process primarily arise from nozzle clogging, unstable ejection pressure, and trajectory deviations. These defects are typically small, irregular in shape, and exhibit low contrast against the surrounding background. Furthermore, ink diffusion exacerbates the visual complexity of the defects. The experimental dataset, provided by a technology company in Shenzhen, China focuses on common defects encountered during the inkjet printing process.

**Figure 1:**
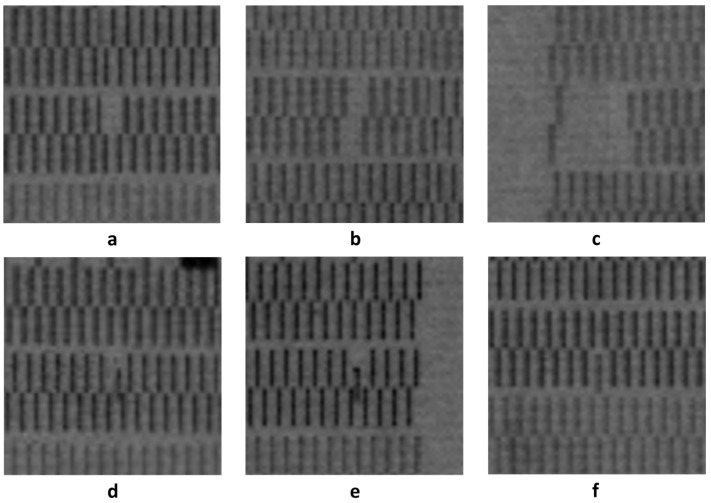
Two types of defects in inkjet datasets: (**a**–**c**) Broken hole (**d**–**f**) Oblique printing.

It includes two key defect types: broken holes and oblique printing. The broken holes defect appears as localized voids within an otherwise continuous printed pattern. The oblique printing defect manifests as noticeable positional deviations in printing columns that should otherwise be aligned regularly. These defect patterns closely correspond to failure modes in semiconductor packaging caused by nozzle clogging, jetting parameter mismatch, and other process fluctuations. Therefore, this dataset serves as an effective benchmark for developing advanced detection algorithms targeting semiconductor manufacturing applications. Figure 1 illustrates representative examples of the two defect types. All images in the dataset were annotated with bounding boxes and class labels using the LabelImg tool, and the defect locations and category information were stored in PASCAL VOC format.

**Figure 2:**
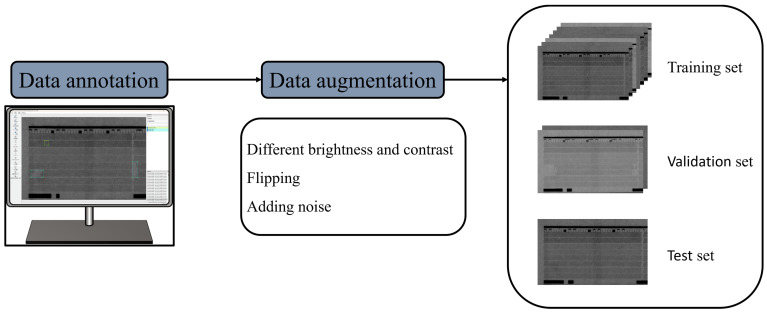
Dataset processing.

To comprehensively represent the complexity of industrial scenarios, the dataset simulates real production conditions, including varying lighting environments and two printing states: high-pressure and atmospheric pressure inkjet. Specifically, three data augmentation methods were employed: adjustment of brightness (−30%) and contrast (−20%) was applied to simulate variations in environmental lighting, image flipping was used to increase dataset diversity, and noise injection (+400%) was introduced to enhance the robustness of the network. The dataset was divided into training, validation, and test sets in a ratio of 7:2:1. The detailed process and category distribution of the dataset are shown in Figure 2 and Table 1.

### 3.2. Proposed MCHB-DETR Detection Model

**Figure 3:**
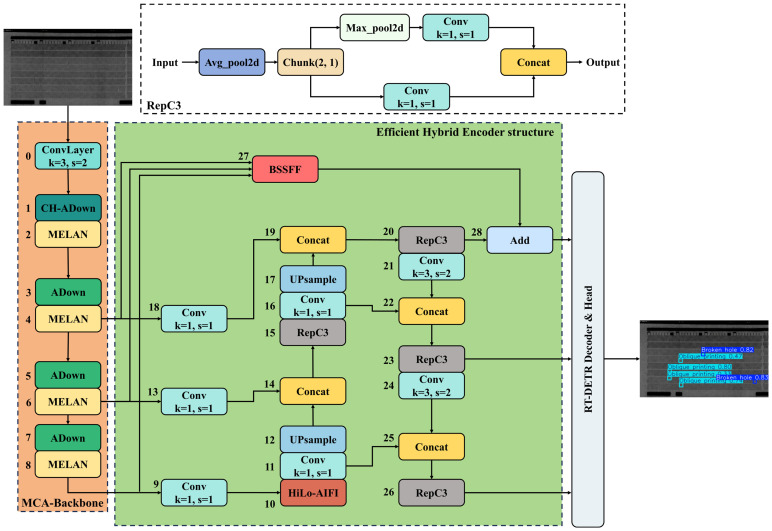
Proposed method framework diagram.

To address the challenges posed by specular reflections and complex background textures in inkjet printing defect detection, we propose a lightweight and efficient multi-scale detection framework, MCHB-DETR (see Figure 3), based on the RT-DETR architecture. This framework incorporates two key enhancements: a compact yet powerful backbone network (MCA-Backbone) designed to improve multi-level feature extraction, and an efficient hybrid encoder capable of capturing cross-frequency and multi-scale information. These components enable the model to better represent small and irregular defects under complex visual conditions. The following sections provide a detailed description of each module’s design.

### 3.3. Layer Aggregation Network MELAN

Classic backbone networks such as ResNet and HGNet have been widely adopted in object detection tasks due to their ability to extract hierarchical features while balancing depth and computational efficiency. ResNet employs residual connections to facilitate gradient propagation in deep architectures, whereas HGNet emphasizes high-resolution representations and cross-stage fusion to better localize fine structures. However, these networks often underperform when handling small, irregular, and texture-mixed defects, such as those encountered in inkjet printing. Specifically, their limited capacity for multi-scale semantic aggregation and channel-wise attention impedes effective feature representation in noisy backgrounds.

In contrast, MobileNet [41] demonstrates that depthwise separable convolutions and inverted residual structures can efficiently capture discriminative feature representations while significantly reducing computational cost, thereby improving the efficiency of deep network layers. Meanwhile, the Efficient Layer Aggregation Network (ELAN) [13] shows that aggregating features across multiple layers effectively enhances feature diversity and representation richness. Inspired by these two design philosophies, we designed the MELAN as part of the MCHB-DETR backbone.

**Figure 4:**
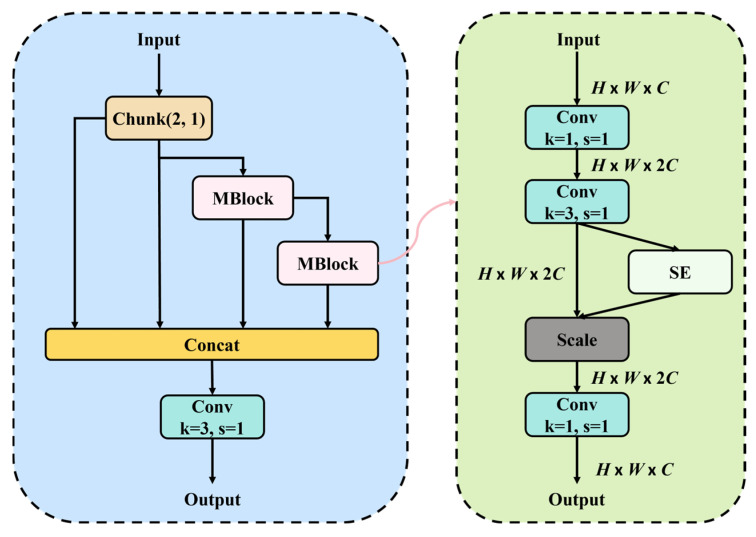
MELAN structure.

Specifically, MELAN utilizes Mobilenet’s inverted residual structure to enhance high-level semantic abstraction, and uses ELAN structure for deep and shallow feature fusion to improve the feature representation ability and semantic diversity of lightweight backbone. At the core of this design is the MBlock, an inverted residual block integrated with an attention mechanism. MBlock aggregates features from different stages of the backbone network, which not only reduces parameter overhead but also enhances the network’s sensitivity to defect-relevant regions. The architecture of this module is illustrated in Figure 4.

Each MBlock first applies a pointwise convolution to expand the channel dimension, enabling a higher-dimensional representation of fine-grained features. This is followed by a 3 × 3 convolution to extract deeper local details. Subsequently, a Squeeze-and-Excitation (SE) module [42] adaptively emphasizes important channels by enhancing their response strengths. Finally, another pointwise convolution compresses the feature map, completing the feature distillation process. Combined with residual connections, this design not only strengthens multi-level feature fusion but also mitigates the risk of gradient vanishing during training. The underlying principle at key stages can be formulated as follows:                 

(1) $$X_{dw}={Conv}_{3\times 3}({Conv}_{1\times 1}\left(X\right))$$

(2) $$s=\sigma (W_{2}\cdot \delta (W_{1}\cdot z))$$

(3) $$X_{n}={Conv}_{1\times 1}(s\cdot X_{dw})$$

(4) $$O=g(X,X_{1},\ldots ,X_{n})$$

Here, $X_{dw}\in R^{H\times W\times tC}$ denotes the feature map obtained after channel expansion and feature extraction, where t is the expansion factor (set to 2 in this work), containing both low-level and high-level feature information. $W_{1}$ and $W_{2}$ are learnable parameters, δ denotes the ReLU activation function, σ denotes the Sigmoid function, and s represents the effective channel-wise weighting ratio learned by the SE module. The resulting feature map is then rescaled through a convolution operation to produce $X_{n}\in R^{H\times W\times C}$, enabling better expression of fine-grained details while maintaining the same spatial resolution as the input, thereby avoiding unnecessary computational overhead in subsequent stages of the model. Finally, the input and the outputs of multiple MBlocks are concatenated and fused through a convolution operation to obtain O, where g denotes the fusion function applied after channe l concatenation.

Compared to traditional CNN layers that follow a strictly sequential and hierarchical structure, MELAN adopts a progressive and cross-connected fusion strategy to integrate multi-level features. This design significantly enhances the robustness of feature extraction for small-scale and texture-blended targets. The resulting backbone output provides richer and more stable representations, serving as a solid foundation for the subsequent encoding and decoding stages.

### 3.4. Downsampling Module

Downsampling is a critical operation in detection backbone networks, used to reduce spatial dimensions and enlarge the receptive field. However, conventional downsampling techniques often lead to the loss of fine structural details. This poses challenges for detecting small, irregular, or low-contrast defects in inkjet printing images.

To address this issue, we introduce the ADown module as a lightweight downsampling alternative to the original backbone design. ADown reduces the spatial resolution of feature maps while preserving more discriminative semantic information compared to conventional downsampling operations, thereby improving feature representation efficiency under limited computational budgets. Nevertheless, when directly applied to inkjet printing images characterized by complex backgrounds and weak defect boundaries, ADown alone remains insufficient to fully preserve defect-sensitive structural cues.

**Figure 5:**
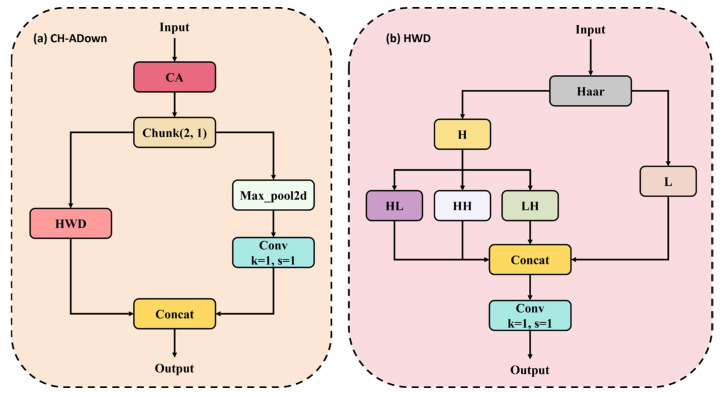
Structure of CH-ADown module.

Motivated by this observation, we further enhance the downsampling process by incorporating CA [43] and Haar wavelet decomposition [44], leading to the proposed CH-ADown module (illustrated in Figure 5). The CA mechanism encodes long-range positional information along both spatial dimensions, enabling the network to focus on defect-relevant regions in cluttered backgrounds. Meanwhile, Haar wavelet decomposition decomposes input features into multiple frequency sub-bands, strengthening edge contours and texture boundaries while suppressing background noise. By integrating these two complementary mechanisms, CH-ADown functions as a structure-aware downsampling module that better preserves critical spatial and frequency information during resolution reduction.

In practice, we employ a dual-stage downsampling strategy in the backbone network. CH-ADown is applied in the early stages to maintain structural fidelity for fine defect patterns, while the simpler ADown module is used in deeper layers to suppress background redundancy and ensure computational efficiency. This progressive design balances accuracy and efficiency, improving the robustness of the backbone under complex inkjet printing conditions.

### 3.5. HiLo-AIFI Module

In Transformer-based object detectors, the self-attention mechanism plays a central role in capturing global dependencies. However, the computational complexity of standard self-attention grows quadratically with respect to the spatial resolution of the feature maps, which severely limits its applicability in real-time, high-resolution defect detection tasks.

**Figure 6:**
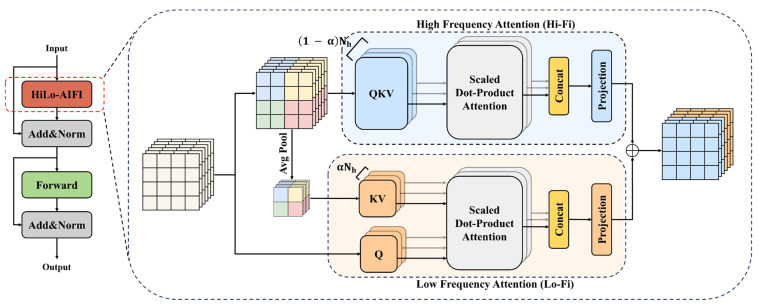
HiLo-AIFI structure.

To address this challenge, we introduce the HiLo-AIFI module [45], as illustrated in Figure 6. As an efficient attention mechanism, it achieves a balance between computational cost and feature representation capability. Inspired by the concept of frequency decomposition, the module is designed to separate low-frequency and high-frequency information for adaptive attention computation. This is effective for detecting defect targets with small size [46].

Specifically, HiLo-AIFI divides the input features into two complementary branches: the Hi branch captures fine-grained, high-frequency local features, which are crucial for delineating edges and small-scale defect patterns. The Lo branch focuses on low-frequency global structures, enabling broader contextual reasoning over a larger receptive field.

Each branch performs attention operations within its respective frequency domain using lightweight formulations. The outputs are then adaptively fused through a gating mechanism, which learns to balance the importance of local and global features based on spatial context. This design enables the module to capture long-range interactions while preserving spatial details, all with reduced computational overhead compared to standard global self-attention.

By embedding HiLo-AIFI into the encoder stage, MCHB-DETR gains the capability to perform efficient and discriminative feature refinement, which is especially beneficial for handling irregular and low-contrast defects commonly encountered in inkjet printing scenarios.

### 3.6. The BSSFF-FPN Network

In industrial defect detection tasks, particularly those involving small-scale or shape-varying targets, effective fusion of features across different spatial resolutions is essential. Traditional Feature Pyramid Networks (FPN) rely on top-down pathways and lateral connections to integrate multi-scale features. However, their performance is often constrained by discrete scale transitions and semantic misalignment, which reduce their ability to model continuous scale variations effectively [47].

**Figure 7:**
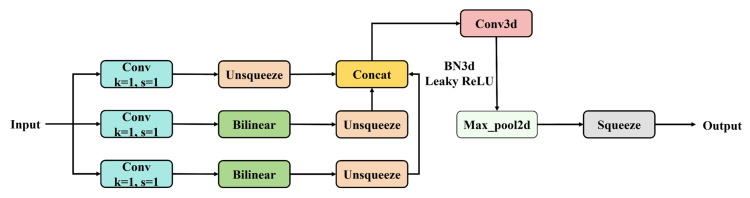
BSSFF structure.

To overcome these limitations, we propose the BSSFF-FPN. This network employs the BSSFF module to enhance the model’s ability to capture and integrate features with diverse scales. This module replaces the upsample module in the SSFF module [48] with bilinear interpolation upsampling without introducing any learning parameters, as illustrated in Figure 7.

BSSFF stacks multi-resolution feature maps from different stages of the backbone network along the scale dimension into a unified 3D tensor. Then, a set of 3D convolutions is applied across the spatial and scale axes to learn contextual dependencies not only within each resolution but also between adjacent scales. This design enables richer contextual information extraction, thereby better capturing and fusing scale-variant features across different resolutions.

Unlike traditional FPNs that perform fusion in a fixed pairwise manner, BSSFF-FPN treats scale fusion as a sequential learning task, enabling more adaptive and flexible information flow. Additionally, batch processing of the feature stacks facilitates efficient parallel computation and continuous scale learning. By embedding BSSFF-FPN into the decoder stage of MCHB-DETR, the model enhances its ability to detect fine-grained defects under complex backgrounds, particularly when faced with size ambiguity or morphological variations.

## 4. Experiments

### 4.1. Experimental Datasets and Evaluation Metrics

**Figure 8:**
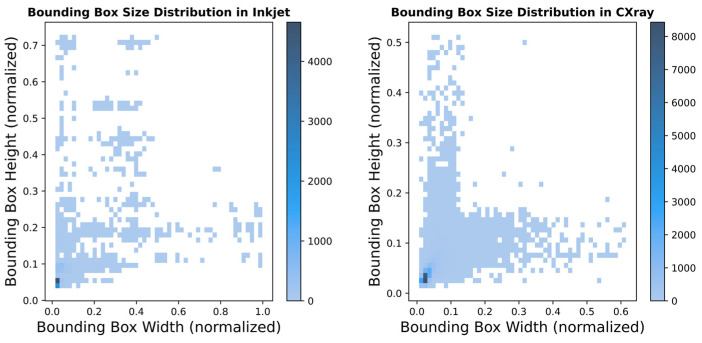
Bounding box distribution of Inkjet and CXray datasets.

To evaluate the effectiveness and generalization capability of the proposed model, we conducted experiments on two datasets from different domains: our inkjet defect dataset and the publicly available CXray industrial defect dataset [49]. Incorporating the latter allows us to assess the model’s adaptability and robustness in cross-domain object detection tasks. Table 2 provides a detailed summary of the specifications of these datasets. Figure 8 provides the case distribution of these datasets.

- The Inkjet dataset is used for inkjet defect detection in this study, containing 2002 real inkjet images with a resolution of 450 × 269 pixels. This dataset covers two typical dynamic defects in the inkjet printing process: broken holes and oblique printing. In the experiments, the dataset was randomly split into training, validation, and testing sets at a ratio of 7:2:1.
- The CXray dataset is employed to verify the generalization capability of the proposed model for small object defect detection. It contains 1960 chip images, each with a resolution of 800 × 600 pixels. These images include over 10,000 bubble defects of various sizes and distributions. Similarly, the dataset was randomly divided into training, validation, and testing sets with a 7:2:1 ratio.

Additionally, based on the two datasets, we employed several evaluation metrics including precision, recall, mean average precision (*mAP*), GFLOPs, parameter count, and frames per second (*FPS*) to compare MCHB-DETR with existing mainstream models.

(5) $$Precision=\frac{TP}{TP\;+\;FP}$$

(6) $$Recall=\frac{TP}{TP\;+\;FN}$$

(7) $$mAP=\frac{\sum_{i=1}^{n}{AP}_{i}}{n}$$

(8) $$FPS=\frac{N}{t}$$

True Positive (*TP*) denotes the number of samples correctly classified as positive, while False Positive (*FP*) refers to the number of samples incorrectly classified as positive. False Negative (*FN*) represents the number of samples mistakenly classified as negative. Precision evaluates the accuracy of the model’s positive predictions, whereas Recall measures the model’s ability to identify all positive samples. mAP comprehensively reflects the interplay between Precision and Recall, indicating the overall detection accuracy of the model. FPS represents the inference speed of the model. Considering model size and practical deployment constraints, the number of parameters and FLOPs are also incorporated as evaluation metrics.

### 4.2. Environment and Training Setup

The experiments were conducted on a platform equipped with an NVIDIA GeForce RTX 4090 GPU and an Intel(R) Xeon(R) Platinum 8352V CPU running at 2.10 GHz, with 24 GB of RAM. The system operated on a 64-bit Windows 10 OS, utilizing PyTorch 2.1.2 as the deep learning framework and Python3.10 for programming, with CUDA 11.8 employed for parallel computing. For comparative experiments, YOLO series and RT-DETR were selected as baseline models. Both detection architectures used models and code versions provided by the Ultralytics team to ensure implementation consistency. The training strategy was uniformly set with an input resolution of 640 × 640 pixels, 250 epochs, and a batch size of 16. The learning rate (lr) and momentum parameters followed Ultralytics’ default adaptive strategy. Additionally, the Adam optimizer was employed to minimize the loss function, ensuring better convergence and higher detection accuracy. To ensure a fair comparison, none of the compared models loaded pretrained weights, and consistent hyperparameters were applied across different datasets. Detailed parameter settings are provided in Table 3.

### 4.3. Ablation Experiments

To evaluate the contribution of each component in the proposed MCHB-DETR, ablation experiments were conducted on the Inkjet dataset. The results are summarized in Table 4. Starting from the baseline RT-DETR-R18, four modules were progressively integrated: ME (MELAN module), CH (CH-Adown and Adown modules), HA (HiLo-AIFI module), and BS (BSSFF module). The experimental results of Models A to D illustrate the impact of each module on overall performance. The ME module significantly improved both performance and efficiency (+0.9% mAP@50, −36.2% FLOPs) by aggregating cross-layer features and enhancing channel responses, thereby boosting representational capacity with fewer parameters and computations. The CH module (+0.7% mAP@50, −7.4% FLOPs), which combines attention-guided downsampling and lightweight convolutions, enhanced the model’s robustness in complex background scenarios, improving detection accuracy under noisy and textured variations. The HA module (+1.0% mAP@50, +1.2% mAP@50:95) strengthened the network’s ability to process frequency-differentiated features, facilitating better detection of irregular targets. The BS module (+0.3% mAP@50, +0.4% mAP@50:95) achieved multi-scale feature continuity and contextual fusion, enabling more effective handling of size ambiguity and morphological variations. Although the enhanced hybrid encoder (Model E) slightly increased model complexity (+2.7% FLOPs, +0.1M parameters), it yielded improvements in mAP (1.8% mAP@50, +1.9% mAP@50:95). The MCA-Backbone, composed of the ME and CH modules (Model F), reduced model complexity (−38% FLOPs, −4.9M parameters) while improving performance (+2.0% mAP@50, +2.2% mAP@50:95). Models F through G gradually integrated all modules, with the fully integrated MCHB-DETR (Model H) achieving increases of +4.5%, +3.3%, +3.1%, and +3.5% in Precision, Recall, mAP@50, and mAP@50:95, respectively, alongside reductions in both parameter count and FLOPs. Each component plays a complementary role in enhancing overall detection performance.

**Figure 9:**
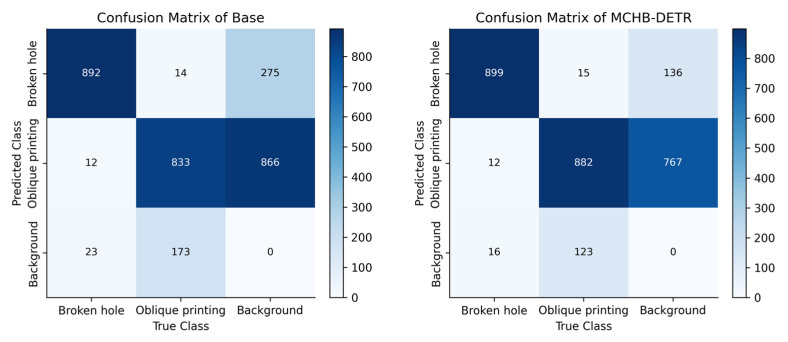
Comparison of confusion matrices.

Figure 9 presents a comparison of the confusion matrices between the baseline model and MCHB-DETR. As shown in the figure, misclassifications mainly occur in the detection of oblique printing defects, which are typically small and visually similar to the background. Compared with the baseline, the proposed MCHB-DETR achieves higher classification accuracy for both defect categories, with particularly notable improvement in detecting oblique printing defects. Moreover, MCHB-DETR effectively reduces misclassification between defect classes and background regions. Although a certain number of false positives still remain, their overall incidence is lower than that of the baseline model, indicating improved discrimination capability under complex background conditions.

The results indicate that MCHB-DETR consistently outperforms the baseline on the Inkjet dataset, in terms of mAP, achieving higher recognition accuracy for broken holes and oblique printing defects while effectively reducing the number of false positives. These findings demonstrate that the proposed method provides tangible improvements in addressing detection challenges arising from complex backgrounds and small-scale defects in inkjet printing processes. It is worth noting that most of the remaining false positives are attributed to background textures with strong contrast or edge patterns that resemble defect structures, suggesting that suppressing visually ambiguous background regions remains a challenging issue for further investigation.

### 4.4. Comparative Experiments with Other Detection Models

To evaluate the performance of MCHB-DETR, we compared the proposed model with several representative methods under unified evaluation metrics across two datasets. The comparison includes Faster R-CNN, Mask R-CNN, Cascade R-CNN and YOLOv5m, YOLOv8m, YOLOv10m, and RT-DETR-R18. Among them, Faster R-CNN, Mask R-CNN, and Cascade R-CNN are all under the MMDtection framework, and flops are measured under 640 × 640 input resolution using inference-only computation. The experimental results demonstrate that the proposed method achieves significant overall improvements.

**Figure 10:**
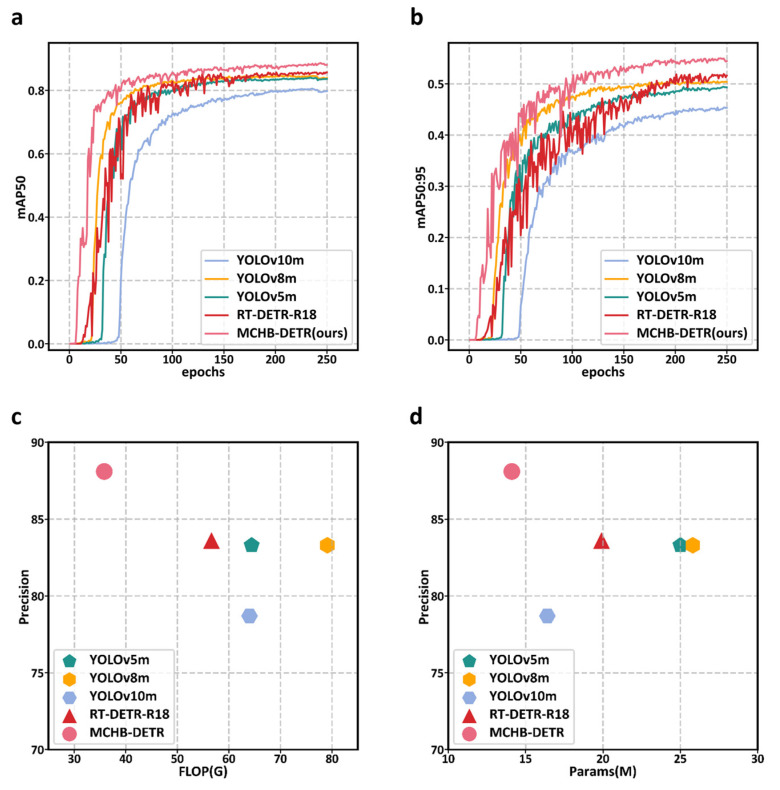
Comparison of model-experiment results on Inkjet dataset: training curves of mAP@50 (**a**) and mAP@50:95 (**b**), and efficiency comparisons in terms of FLOPs (**c**) and parameter counts (**d**).

The results on the Inkjet dataset are presented in Table 5. The training results of some models are shown in Figure 10. MCHB-DETR achieves a precision of 88.1%, recall of 83.0%, mAP@50 of 88.6%, mAP@50:95 of 55.1%, and an inference speed of 275.2 FPS, while also significantly reducing the number of parameters and FLOPs compared to other methods. These findings indicate that the proposed method outperforms other approaches in terms of overall performance on the Inkjet dataset. This improvement can be attributed to the lightweight backbone, which enables efficient feature extraction, and its coupling with the enhanced hybrid encoder. This synergy allows the model to accurately capture the morphological characteristics of defects while meeting real-time detection requirements.

**Figure 11:**
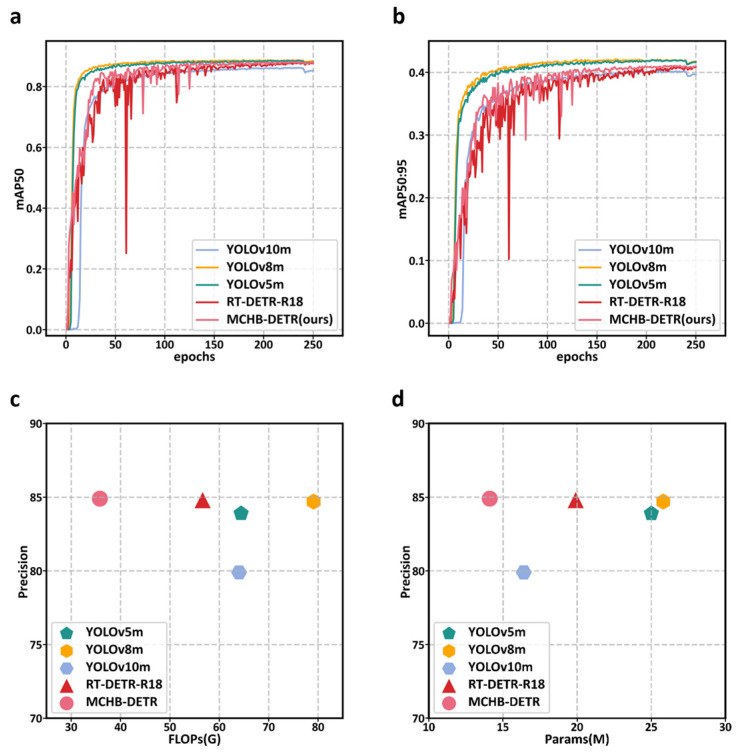
Comparison of model-experiment results on CXray dataset: training curves of mAP@50 (**a**) and mAP@50:95 (**b**), and efficiency comparisons in terms of FLOPs (**c**) and parameter counts (**d**).

The experimental results of MCHB-DETR on the CXray dataset are shown in Table 6 and Figure 11. The results indicate that the model maintains comparable detection performance to RT-DETR-R18, achieving a precision of 84.9%, recall of 82.1%, mAP@50 of 88.0%, and mAP@50:95 of 41.1%, while exhibiting significant computational efficiency. Specifically, MCHB-DETR requires only 14.1M parameters and 35.8 GFLOPs, which are substantially lower than those of the compared methods. This demonstrates that MCHB-DETR is more computationally efficient and capable of operating with significantly reduced computational resources.

The performance discrepancy observed between the Inkjet dataset and the CXray dataset can be attributed to the distinct characteristics of the two inspection scenarios. The CXray dataset is associated with relatively simple background conditions and clearer defect patterns. Under such circumstances, standard detectors such as RT-DETR already achieve strong baseline performance, leaving limited room for further improvement. In contrast, the Inkjet dataset contains more complex backgrounds, low-contrast defects, and irregular defect morphologies, where the proposed MCHB-DETR is able to better exploit its architectural advantages.

Notably, despite the limited performance margin on the CXray dataset, MCHB-DETR maintains competitive detection accuracy without introducing performance degradation, demonstrating its robustness and general applicability across different industrial inspection tasks.

**Figure 12:**
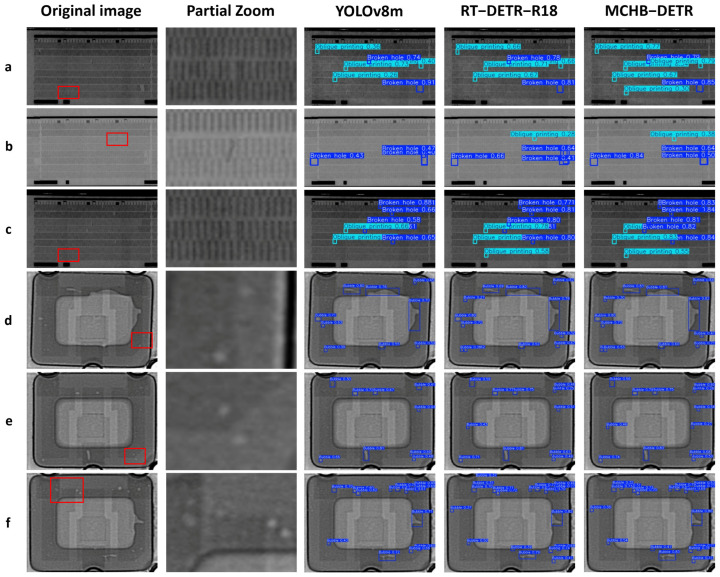
Comparison of detection results on different datasets. (**a**–**c**) Inkjet (**d**–**f**) CXray. Partial Zoom is a magnified image of the red area.

To verify the practicality and robustness of the proposed model in real-world applications, we utilized the test sets from both the Inkjet and CXray datasets to simulate deployment scenarios in actual industrial environments. As illustrated in Figure 12, the “Partial Zoom” represents a magnified view of the red box area in the “Original Image.” From the detection results on the Inkjet dataset, it is evident that YOLOv8m and RT-DETR-R18 exhibit notable false positives and missed detections when identifying subtle edge variations in inkjet defects. Their overall detection performance remains limited and falls short of meeting the accuracy requirements for inkjet defect inspection. In contrast, the proposed model demonstrates superior performance in the detection task, achieving nearly complete target localization. Similarly, on the CXray dataset, although MCHB-DETR shows occasional false positives, it still exhibits strong capability in detecting small bubble defects. Notably, the model successfully identifies defects even in high-density defect regions.

### 4.5. Comparative Experiments with Other Backbone Structure

**Figure 13:**
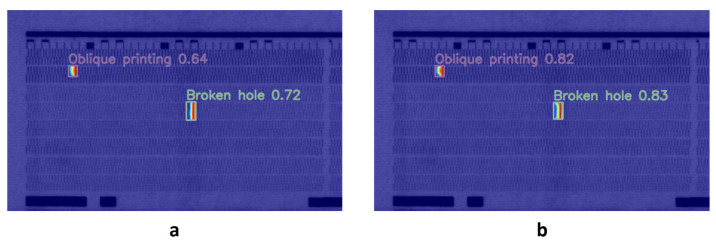
Comparison of heat map visualization: (**a**) R18 (**b**) MCA.

To validate the architectural superiority of MCA (MCA-Backbone) in inkjet defect detection tasks, we compared three backbone networks: R34 (ResNet34), R50 (ResNet50), and HG (HGNet). R34 and R50 adopt BasicBlock and Bottleneck modules, respectively, to construct hierarchical residual structures, extracting features through stacked standard 3 × 3 convolutions. HG, leveraging a multi-branch parallel architecture and dense cross-hierarchical connections, has demonstrated strong competitiveness in industrial inspection tasks. The experimental results are presented in Table 7. As shown, despite utilizing fewer computational resources, the MCA-Backbone still achieves superior performance in terms of detection accuracy, with a mAP50 of 87.5% and a mAP50-95 of 53.8%. This is attributed to its ability to construct a high-dimensional feature space via layer aggregation networks and to effectively fuse and extract features through a frequency-spatial collaborative downsampling strategy, thereby enhancing the model’s capability to detect defective targets. Additionally, we compared the visualization results of two different backbone networks, R18 and MCA (Figure 13). The heatmaps indicate that MCA significantly enhances the confidence level in inkjet defect detection, thereby demonstrating the effectiveness of the proposed method.

### 4.6. Robustness Comparison Experiment

**Figure 14:**
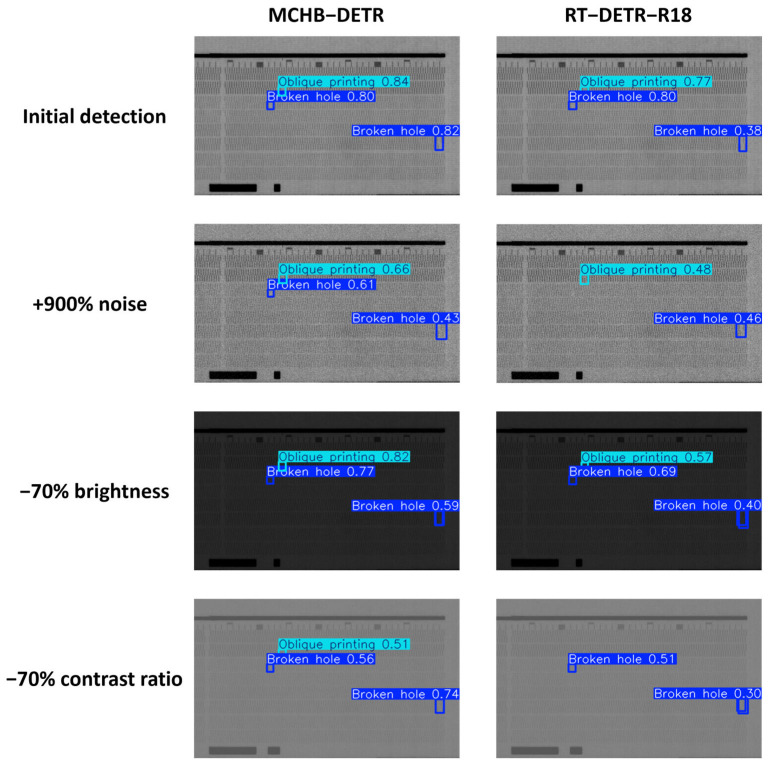
Comparison of Robustness between MCHB-DETR and R18.

To simulate a real production environment and assess the robustness of MCHB-DETR, we altered the image quality by increasing noise levels and reducing brightness and contrast. The experimental results are shown in Figure 14. As shown, the proposed method successfully detects the corresponding targets even under lower image quality. In contrast, the original model fails to detect targets after reducing contrast and increasing noise levels, and produces repeated detections when brightness is reduced. These results highlight the robustness of the method.

## 5. Conclusions and Future Work

This work proposes MCHB-DETR, a lightweight deep learning framework improved from RT-DETR, specifically designed for detecting inkjet printing defects in semiconductor packaging processes. The framework employs the MELAN, which strengthens defect detail representation while significantly reducing the number of parameters and computational complexity, making it more suitable for edge deployment on production lines. By incorporating CH-Adown and Adown downsampling strategies, the model effectively preserves fine edge structures that are crucial for maintaining circuit continuity. The introduced hybrid encoder, composed of the HiLo-AIFI and BSSFF modules, enables the model to jointly capture high frequency details and low frequency contextual information, thus achieving robust multi scale defect detection under complex backgrounds and substantially improving the identification of critical defects such as short circuits and open circuits.

Experiments conducted on both the inkjet printing dataset and the CXray industrial defect dataset demonstrate that MCHB-DETR achieves a favorable balance between detection accuracy and inference speed, exhibiting strong cross domain generalization capability. This provides a reliable solution for high precision and high efficiency visual inspection in semiconductor packaging.

However, current research still faces several limitations. The dataset size remains insufficient to support the deep generalization required for effective model training. In complex background scenarios, false positives occur frequently, and the model’s real-time detection capability still lags state-of-the-art models such as YOLOv5, YOLOv8, and YOLOv10. To address these challenges, future work will focus on integrating unsupervised learning with adversarial networks to improve the model’s generalization ability. Furthermore, the model architecture will be refined to balance performance and size, thereby enhancing overall robustness and applicability.

## Figures and Tables

**Table 1:** Distribution of Defect Categories in Inkjet Dataset.

Defect Type	Defect Count			
	Train	Val	Test	Total
Broken hole	3513	927	506	4946
Oblique printing	3945	1020	533	5498

**Table 2:** Detailed information on the Inkjet, CXray datasets.

Dataset	Defect Type	Total Image	Image Size	Resource
Inkjet	2	2002	450 × 269	-
CXray	1	1960	800 × 600	https://github.com/EudicL/CXray (accessed on 13 May 2025)

**Table 3:** Parameters settings.

Parameters	Configuration
Epochs	250
Lr0	0.001
Batch size	16
momentum	0.937
optimizer	Adam

**Table 4:** Ablation experiments results for MCHB-DETR.

Model	ME	CH	HA	BS	Params (M)	FLOPs (G)	Precision	Recall	mAP_50_	mAP_50:95_
Base					19.9	56.6	83.6	79.6	85.5	51.6
A	√				14.6	36.1	84.1	80.7	86.4	52.9
B		√			17.5	52.4	85.7	79.6	86.2	52.6
C			√		19.9	56.6	86.2	79.6	86.5	52.8
D				√	20.0	58.0	85.3	78.4	85.8	52.0
E			√	√	20.0	58.1	84.8	81.9	87.3	53.5
F	√	√			14.1	**35.1**	87.2	81.6	87.5	53.8
G	√	√	√		**14.0**	**35.1**	87.9	82.0	88.1	54.4
H	√	√	√	√	14.1	35.8	**88.1**	**82.9**	**88.6**	**55.1**

The symbol indicates the usage of the corresponding module. Bold indicates the optimal data in the corresponding column.

**Table 5:** Comparison experiments of different models on Inkjet dataset.

Model	Params (M)	FLOPs (G)	Precision	Recall	mAP_50_	mAP_50–95_	FPS
Faster R-CNN	41.4	90.9	82.0	81.3	83.6	47.1	48.5
Mask R-CNN	43.9	142.0	86.3	75.8	82.9	48.7	46.7
Cascade R-CNN	69.2	119.0	80.9	80.3	83.7	47.3	41.3
YOLOv5m	25.0	64.4	83.3	75.7	83.6	49.3	**485.8**
YOLOv8m	25.8	79.1	83.3	78.7	84.5	50.4	422.1
YOLOv10m	16.4	64	78.7	72.4	80.1	45.4	403.0
RT-DETR-R18	19.9	56.6	83.6	79.6	85.5	51.6	174.7
MCHB-DETR	**14.1**	**35.8**	**88.1**	**83.0**	**88.6**	**55.1**	275.2

Bold indicates the optimal data in the corresponding column.

**Table 6:** Comparison experiments of different models on CXray dataset.

Model	Params (M)	FLOPs (G)	Precision	Recall	mAP_50_	mAP_50–95_
Faster R-CNN	41.3	90.9	80.9	74.9	80.4	37.3
Mask R-CNN	44.0	142.0	83.4	75.3	81.3	38.6
Cascade R-CNN	69.2	119.0	83.5	75.0	81.4	38.7
YOLOv5m	25.0	64.4	83.7	**82.5**	88.5	42.0
YOLOv8m	25.8	79.1	84.2	82.2	**88.6**	**42.1**
YOLOv10m	16.4	64.0	81.5	78.5	86.1	40.2
RT-DETR-R18	19.9	56.6	84.8	82.0	87.8	40.9
MCHB-DETR	**14.1**	**35.8**	**84.9**	82.1	88.0	41.1

Bold indicates the optimal data in the corresponding column.

**Table 7:** Comparison experiments of different backbone frameworks.

Model	R34	R50	HG	MCA	Params (M)	FLOPs (G)	mAP_50_	mAP_50:95_
RT-DETR	√				37.2	107.7	87.4	52.3
		√			42.7	130.5	87.6	51.9
			√		32.8	108	**87.7**	52.1
				√	**14.1**	**35.1**	87.5	**53.8**

The symbol indicates the usage of the corresponding module. Bold indicates the optimal data in the corresponding column.

## Data Availability

Data are available on reasonable request from the corresponding authors.
